# Retraction Note: Effect of radiotherapy on head and neck cancer tissues in patients receiving radiotherapy: a bioinformatics analysis-based study

**DOI:** 10.1038/s41598-025-98250-2

**Published:** 2025-04-28

**Authors:** Zhenjie Guan, Jie Liu, Lian Zheng

**Affiliations:** 1 Department of Stomatology, The First Affiliated Hospital of Zhengzhou University, Zhengzhou, 450052 Henan China; 2 Department of Oral and Maxillofacial Surgery, The First Affiliated Hospital of Zhengzhou University, NO.1 Jianshedong Road, Zhengzhou, 450052 Henan China

Retraction of: *Scientific Reports* 10.1038/s41598-024-56753-4, Published online 15 March 2024

The Editors have retracted this Article. After publication, concerns were raised about the validity of the research presented in this Article. One of the databases used by the Authors in their research, the Cancer Genome Atlas–Head-Neck Squamous Cell Carcinoma (TCGA-HNSC), does not involve tissue samples from patients subject to radiotherapy, contrary to what is reported in this Article.

The Authors provided the TCGA-HNSC IDs of the samples analysed to the Editors. However, the Editors confirmed that the TCGA-HNSC records show that the samples were collected prior to the start of any adjuvant treatment. The Editors therefore no longer have confidence in the analysis and results reported in this Article.

Lian Zheng has not stated whether they agree or disagree with this retraction. Zhenjie Guan and Jie Liu have not replied to correspondence from the Editors.

